# Maternal Blood Pressure During Pregnancy and Early Childhood Blood Pressures in the Offspring: The GUSTO Birth Cohort Study: Erratum

**DOI:** 10.1097/01.md.0000479948.71542.ff

**Published:** 2016-01-22

In the article “Maternal Blood Pressure During Pregnancy and Early Childhood Blood Pressures in the Offspring: The GUSTO Birth Cohort Study”,^1^ which appeared in Volume 94, Issue 45 of *Medicine*, Dr. Ngee Lek's name was incorrectly presented as Lek Ngee. The article has since been corrected online.
